# Attitudes toward sports, academic motivation, and academic life satisfaction: a cross-sectional analysis of indirect associations

**DOI:** 10.3389/fpsyg.2026.1906871

**Published:** 2026-07-28

**Authors:** Yaşar İsmail GüIünay

**Affiliations:** Hasan Doğan Faculty of Sports Sciences, Karabük University, Karabük, Türkiye

**Keywords:** academic life satisfaction, academic motivation, attitudes toward sports, higher education, self-determination theory

## Abstract

**Background:**

Academic life satisfaction is an important indicator of students' experiences in higher education, yet the mechanisms linking sport-related attitudes to academic life remain unclear. Guided by Self-Determination Theory, this study examined the associations among attitudes toward sports, academic motivation, and academic life satisfaction, with particular attention to the role of academic motivation as a statistical linking variable.

**Methods:**

A cross-sectional correlational design was employed with 434 undergraduate students from non-sport academic programs at a public university in Türkiye. Participants completed measures of attitudes toward sports, academic motivation, and academic life satisfaction. The hypothesized indirect association model was tested using Hayes' PROCESS Model 4 with bootstrap procedures.

**Results:**

Positive attitudes toward sports were associated with higher academic motivation and greater academic life satisfaction. Academic motivation was also positively associated with academic life satisfaction. Although attitudes toward sports were initially related to academic life satisfaction, this direct association became non-significant when academic motivation was included in the model. The indirect association involving academic motivation was statistically significant.

**Conclusion:**

The findings suggest that the relationship between attitudes toward sports and academic life satisfaction is better understood when academic motivation is taken into account. Rather than exerting a direct association, positive attitudes toward sports may be related to academic life satisfaction through motivational processes associated with learning. Although causal conclusions cannot be drawn from the cross-sectional design, the findings highlight the importance of motivation in understanding how students' orientations toward sport are related to their academic experiences.

## 1 Introduction

Higher education is not only a period in which individuals acquire knowledge and professional skills, but also a formative context in which self-perceptions are shaped, autonomy is strengthened, and life goals are re-evaluated (Komarraju et al., 2010; Pascarella and Terenzini, 2005). For this reason, evaluating students' university experiences solely in terms of academic performance risks overlooking important dimensions of student development, including psychological wellbeing, social adjustment, and perceptions of the learning environment (Institute, 2024). Increasingly, higher education is understood as a setting that should support not only academic achievement, but also students' emotional, social, and cognitive development (Yükseköğretim Kurulu, 2023). Within this broader perspective, academic life satisfaction has become an important concept for understanding students' university experiences in a more holistic way. Academic life satisfaction refers to students' overall evaluation of their academic experiences, including the learning process, the educational environment, and their interactions within academic settings (Guevara et al., 2025; Mazzeo et al., 2025; Nogueira et al., 2019; Schnettler et al., 2017). Previous research has shown that academic life satisfaction is closely related to academic achievement, psychological resilience, and general life satisfaction (Bagdžiuniene et al., 2025; Ferreira and Teixeira, 2021; Çivitçi, 2012; Oliveira and Marques, 2024). Students with higher levels of academic life satisfaction tend to report stronger academic engagement, greater self-efficacy, and more positive educational experiences, whereas lower levels of satisfaction have been linked to disengagement, academic burnout, and emotional difficulties (Çivitçi, 2012; Morelli et al., 2023; Oliveira and Marques, 2024). These findings suggest that academic life satisfaction is not merely an outcome of academic success, but also an important indicator of how students experience university life more broadly. One of the factors most consistently associated with positive academic experiences is academic motivation. Academic motivation reflects the willingness to learn, persist, achieve, and invest effort in educational activities (Vallerand et al., 1992). In higher education, motivation plays a central role in shaping how students engage with learning, respond to academic demands, and evaluate their educational experiences. Students who perceive learning as meaningful, feel competent in their academic work, and act with a sense of autonomy are more likely to benefit from the learning process and report higher levels of academic satisfaction (Morelli et al., 2023; Mistry, 2024; Ryan and Deci, 2006; Yoo and Marshall, 2022). In this respect, academic motivation can be seen not only as a driver of academic behavior, but also as an important psychological resource associated with how positively students experience academic life.

Motivation has often been studied in relation to classroom practices, teacher-student relationships, and the broader learning environment (Niemiec and Ryan, 2009; Reeve, 2012). However, motivational processes are not shaped only within formal academic settings. Lifestyle-related habits and behavioral orientations may also influence how students approach learning and derive meaning from their academic experiences (Jeno et al., 2018). While academic satisfaction primarily focuses on students' contentment with specific educational programs and coursework (Morelli et al., 2023; Mistry, 2024), academic life satisfaction, as addressed here, reflects a broader cognitive evaluation of the entire university experience, including social integration and campus environment (Nogueira et al., 2019; Eccles and Barber, 1999; Sentürk, 2019). Crucially, it is important to distinguish that positive attitudes toward sports reflect a psychological orientation and a set of values regarding physical engagement, which may not always equate to high-intensity physical activity participation. However, from a psychological perspective, these attitudes represent a potential reservoir of personal resources—such as goal-setting tendencies and resilience—that may independently influence students' academic approach. This suggests that the factors supporting academic motivation may extend beyond the classroom and include students' broader patterns of daily life and engagement. One such factor may be students' attitudes toward sports. Some studies suggest that healthy lifestyle behaviors, including participation in sports and physical activity, may contribute to academic motivation and satisfaction by supporting wellbeing, self-regulation, and personal discipline (Edens, 2008; Özenoglu et al., 2024). More broadly, sport has been associated not only with physical health, but also with psychosocial processes that may be relevant to students' academic experiences. Positive attitudes toward sports have been linked to self-efficacy, stress management, resilience, and social support, all of which may be relevant to students' academic experiences (Aizava et al., 2024; Han, 2025a,b; Kan and Han, 2025; Koçak, 2014; Özcan et al., 2025; Özenoglu et al., 2024). Research on university students has also shown that physical activity and sport participation are positively associated with academic success, attention, and motivation (Choi et al., 2024; Fredricks and Eccles, 2006; Leong et al., 2018; Delito, 2023; Gülünay and Savas, 2022; Iqbal et al., 2020; Sasongko et al., 2025). Taken together, these findings suggest that students' orientations toward sports may have implications that extend beyond physical activity itself and may also be relevant to their academic lives.

The present study is grounded in Self-determination Theory (SDT), which serves as a theoretical lens to interpret how students' broader orientations—such as those toward sports—may satisfy basic psychological needs and consequently foster academic motivation (Connault, 2010; Ryan and Deci, 2000, 2008). While SDT typically focuses on the satisfaction of autonomy, competence, and relatedness, this study utilizes the theory to contextualize how positive sport-related attitudes might act as a precursor to the motivational resources necessary for academic life satisfaction (Kusurkar et al., 2013; Orsini et al., 2015). By viewing sports as a context that can potentially satisfy these needs, we can better understand the pathways through which extra-curricular orientations manifest within the academic domain. Despite the growing body of research on academic motivation, academic life satisfaction, and the role of sport and physical activity, these variables have often been examined separately. Furthermore, while existing literature often conflates physical activity participation with attitudes toward sport, our approach highlights the independent predictive power of students' internal attitudes in shaping the motivational landscape of their university life. Relatively limited attention has been given to whether attitudes toward sports are indirectly associated with academic life satisfaction through academic motivation within a single analytical framework. Addressing this gap may contribute to a more integrated understanding of how students' orientations toward sport are related to their academic experiences. Accordingly, the present study examines the associations among students' attitudes toward sports, academic motivation, and academic life satisfaction. More specifically, it evaluates whether academic motivation statistically accounts for the indirect association between attitudes toward sports and academic life satisfaction. The model is grounded in Self-Determination Theory as an interpretive framework and is tested using cross-sectional correlational data. Therefore, the proposed model should be understood as a theory-informed statistical model of indirect association rather than as evidence of temporal or causal mediation. Based on the theoretical framework and previous empirical findings, the following hypotheses were formulated.

**H1:** Attitudes toward sports are positively associated with academic motivation.

**H2:** Attitudes toward sports are positively associated with academic life satisfaction.

**H3:** Academic motivation is positively associated with academic life satisfaction.

**H4:** Academic motivation statistically accounts for the indirect association between attitudes toward sports and academic life satisfaction.

**Figure 1:**
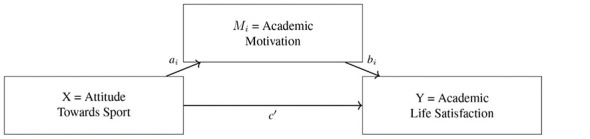
Conceptual indirect association model illustrating the relationships among attitudes toward sports, academic motivation, and academic life satisfaction.

The conceptual model reflecting the hypothesized relationships among the variables is presented in Figure 1.

## 2 Material and method

### 2.1 Research model

The primary aim of this study is to examine the associations among students' attitudes toward sports, academic motivation, and academic life satisfaction within a higher education context. In addition to bivariate associations, a theory-informed indirect association model grounded in Self-determination Theory was specified to evaluate whether academic motivation statistically accounts for the association between attitudes toward sports and academic life satisfaction (Deci and Ryan, 2000; Ryan and Deci, 2017). The study employed a cross-sectional, quantitative, correlational research design (Karasar, 2020). This design enables the examination of patterns of association among variables measured at a single point in time but does not permit conclusions regarding temporal ordering or causal relationships. Accordingly, the model tested in this study should be interpreted as a statistical model of indirect association rather than as evidence of an underlying causal mediation process (Hayes, 2022; MacKinnon et al., 2004). More specifically, the analyses were designed to estimate direct, indirect, and total associations among the study variables, based on theoretically informed assumptions derived from Self-determination Theory (Deci and Ryan, 2000; Ryan and Deci, 2017). While the proposed model is consistent with a directional conceptual framework in which attitudes toward sports are associated with academic motivation, which in turn is associated with academic life satisfaction, the cross-sectional nature of the data precludes inferences about causality or temporal precedence. Therefore, the findings are interpreted in terms of associational patterns and the extent to which academic motivation may statistically account for the relationship between attitudes toward sports and academic life satisfaction (Hayes, 2022).

### 2.2 Participants

The participants in this study consisted of undergraduate students enrolled in different faculties at a public university in Türkiye. A non-probability sampling approach was adopted, combining predefined eligibility criteria with voluntary participation. To minimize potential bias associated with students whose academic training is directly centered on sport, individuals enrolled in sport-related programs were intentionally excluded from the sampling frame. Sport-related programs were defined based on official university categorization, specifically targeting departments housed within the Faculty of Sports Sciences (e.g., Physical Education, Sports Management, and Recreation). This operational definition ensures that sport-related attitudes are distinct from formal professional academic training. This decision was made to ensure that attitudes toward sports could be examined within a broader higher education context, among students for whom sport does not constitute the central focus of their academic training. Data were initially collected from 441 students. During the data screening process, incomplete or otherwise unusable responses were excluded, resulting in a final analytic sample of 434 participants. Of the 441 initial responses, seven were deemed unusable: four due to missing data (exceeding 10%) and three due to systematic response patterns (e.g., identical mid-point responses across the entire scale). Of these, 234 were female (53.9%) and 200 were male (46.1%), with a mean age of 21.37 years (SD = 2.73). The demographic characteristics of the participants are presented in Table 1. Participants were recruited via convenience sampling across five non-sport-related faculties: engineering, Education, Economics, Arts and Sciences, and Theology. Instructor permission was explicitly granted for data collection during 15 mandatory undergraduate core courses. Regarding the university context, the institution provides standard athletic facilities and hosts university-sponsored sports teams (e.g., football, volleyball, and basketball) accessible to all students. Given that the sample was drawn exclusively from non-sport-related disciplines, the university's general sports reputation served as a neutral environmental background rather than a confounding academic factor.

**Table 1:** Demographic characteristics of the study participants.

Gender	*n*	%	
Female	234	53.9	
Male	200	46.1	
Total	434	100.0	
	***N***	***M***	**SD**
Age	434	21.37	2.73

### 2.3 Data collection process

Data were collected through face-to-face, paper-based questionnaire administration between September 1 and 19, 2025. The questionnaires were administered in classroom settings at the university on a voluntary basis. During the administration process, the researcher was present in the classroom, and participants completed the questionnaires individually under supervised conditions. To support response consistency and reduce potential peer influence, students were asked to complete the forms independently during the session. The completion of the questionnaire package took approximately 10–15 min.

### 2.4 Data collection tools

#### 2.4.1 Attitudes toward sport scale

Attitudes toward sports were measured using the Attitudes Toward Sport Scale developed in Turkish by Koçak (2014). The scale consists of 22 items rated on a 5-point Likert scale and is designed to assess students' overall attitudes toward sports. Total scores range from 22 to 110, with higher scores indicating more positive attitudes toward sports. In the original scale development study, the internal consistency coefficient for the overall scale was reported as 0.89. In the present study, Cronbach's alpha for the total scale was 0.94.

#### 2.4.2 Academic motivation scale

Academic motivation was assessed using the Academic Motivation Scale developed by Vallerand et al. (1992) and adapted into Turkish by Karagüven Ünal (2012). The original scale consists of 28 items rated on a 7-point Likert scale ranging from 1 (“strongly disagree”) to 7 (“strongly agree”) and includes subdimensions reflecting intrinsic motivation, extrinsic motivation, and amotivation. Previous research has indicated that the amotivation dimension reflects the absence of motivation and may show a negative or near-zero association with the overall scale score. Accordingly, when a global academic motivation score is calculated, it is recommended that the amotivation items be excluded to avoid conceptual inconsistency (Karagüven Ünal, 2012). In line with this recommendation, the four items representing amotivation were excluded in the present study, and analyses were conducted based on the remaining 24 items. Total scores therefore ranged from 24 to 168, with higher scores indicating higher levels of academic motivation. The internal consistency coefficient (Cronbach's alpha) for the scale was reported as 0.81 in the original study and 0.87 in the Turkish adaptation. In the present study, the Cronbach's alpha coefficient was 0.93, indicating high internal consistency.

#### 2.4.3 Academic life satisfaction scale

Academic life satisfaction was measured using the Academic Life Satisfaction Scale developed by Nogueira et al. (2019) and adapted into Turkish by Odaci et al. (2021). The scale consists of 8 items rated on a 5-point Likert scale ranging from 1 (“strongly disagree”) to 5 (“strongly agree”) and is designed to assess students' overall satisfaction with their academic experiences, including their engagement with the learning process and their perceptions of the academic environment. Total scores range from 8 to 40, with higher scores indicating greater academic life satisfaction. The internal consistency coefficient (Cronbach's alpha) was reported as 0.80 in the original study and 0.86 in the Turkish adaptation. In the present study, the Cronbach's alpha coefficient was 0.78, indicating acceptable internal consistency.

### 2.5 Research ethics

This study was conducted in accordance with the ethical principles for research involving human participants and the Declaration of Helsinki. Ethical approval was obtained from the Social and Human Sciences Research Ethics Committee of Karabük University (Meeting no: 2025/05, Decision no: 85, May 30, 2025) prior to data collection. Before participation, students were informed about the purpose of the study, the voluntary nature of participation, their right to withdraw at any stage without penalty, and the confidential handling of the data. Written informed consent was obtained from all participants. To protect participant privacy, no personally identifying information was collected, and all responses were processed anonymously.

### 2.6 Data analysis

Data were analyzed using IBM SPSS Statistics (Version 27), AMOS, and the PROCESS macro (Version 4.2). Prior to the main analysis, the dataset was screened for missing values and outliers, and only complete and valid responses were retained for further analysis. To evaluate the suitability of the data for parametric analyses, the distributional properties of the variables were examined using skewness and kurtosis coefficients (Hair et al., 2019). Internal consistency was assessed using Cronbach's alpha coefficients (George and Mallery, 2003), and the structural validity of the measurement instruments was examined through confirmatory factor analysis (CFA) using the Maximum Likelihood estimation method (Byrne, 2016; Kline, 2023). To address the potential for common method bias (CMB) inherent in self-report studies, a Harman's single-factor test was conducted as a procedural and statistical check (Podsakoff et al., 2003). All measurement items were entered into an unrotated exploratory factor analysis, and the first factor accounted for 22.83% of the total variance. As this value was substantially below the commonly accepted threshold of 50%, common method bias was not considered a serious concern in the present study. Additionally, a CFA-based comparison was conducted between our proposed multi-factor measurement model and a single-factor model, in which all measurement items were constrained to load onto a single latent factor. The single-factor model yielded a poor fit to the data [*x*^2^ (1,377) = 7,935, *CFI* = 0.426, *TLI* = 0.404, *RMSEA* = 0.105], which was significantly worse than our proposed measurement model, further supporting the distinctiveness of our study constructs. After the psychometric properties of the instruments had been evaluated, Pearson correlation coefficients were calculated to examine the bivariate associations among attitudes toward sports, academic motivation, and academic life satisfaction. To test the hypothesized indirect association, a regression-based path model was estimated using Hayes' PROCESS Model 4 with 5,000 bootstrap resamples and 95% confidence intervals (Hayes, 2022). In this model, gender and age were included as covariates to control for their potential influence on the associations among the study variables. In line with established recommendations, an indirect association was considered statistically significant when the bootstrap confidence interval did not include zero (Hayes, 2022; MacKinnon et al., 2004). An a priori power analysis was conducted using G^*^Power 3.1 (Faul et al., 2007). Using the *F*-test family and the statistical test “Linear multiple regression: fixed model, *R*^2^ deviation from zero,” with a medium effect size (*f* ^2^ = 0.15), α = 0.05, statistical power (1–β) = 0.95, and three predictors, the required minimum sample size was approximately 119 participants. Given that the present study included 434 participants, the sample size was considered more than adequate for the planned regression analyses. It should be noted that this analysis pertains to the regression components of the model and does not constitute a direct power analysis of the bootstrapped indirect effect. Because the data were cross-sectional, the indirect effect estimates were interpreted as statistical evidence of indirect association rather than as evidence of causal mediation.

### 2.7 Measurement validity and reliability

To evaluate the psychometric properties of the measurement instruments, factor loadings, Composite Reliability (CR), and Average Variance Extracted (AVE) values were examined. Factor loadings ranged from 0.48 to 0.74 for the Attitudes Toward Sports Scale, from 0.31 to 0.76 for the Academic Motivation Scale, and from 0.49 to 0.76 for the Academic Life Satisfaction Scale. Although one item in the Academic Motivation Scale showed a relatively low standardized factor loading (0.31), it was retained because the scale is an established and previously validated instrument, and removing the item would have altered its original factorial structure. Moreover, the overall composite reliability of the scale remained satisfactory (CR = 0.91), supporting the retention of all 24 items. CR values ranged between 0.85 and 0.94, indicating satisfactory internal consistency. Although AVE values were below the conventional 0.50 threshold for all constructs, CR values exceeded 0.70, suggesting that convergent validity was only partially supported. According to Fornell and Larcker (1981), acceptable composite reliability may partially compensate for lower AVE values; however, the convergent validity findings should be interpreted with caution (Hair et al., 2019). The psychometric characteristics of the measurement instruments are presented in Table 2.

**Table 2:** Factor loading, reliability, and convergent validity results.

Scale	Items	Factor loadings	AVE	CR
Attitudes toward sports	22	0.48–0.74	0.41	0.94
Academic motivation	24	0.31–0.76	0.32	0.91
Academic life satisfaction	8	0.49–0.76	0.42	0.85

CR, Composite Reliability; AVE, Average Variance Extracted. Although AVE values were below the recommended threshold of 0.50, CR values exceeded 0.70 for all constructs. Therefore, convergent validity was considered to be only partially supported and should be interpreted with caution (Fornell and Larcker, 1981; Hair et al., 2019).

## 3 Results

This section presents the results of the analyses conducted in the study. Before proceeding to the main analyses, the psychometric properties of the measurement instruments were evaluated and are presented in Table 2.

As it is seen in Table 3; skewness and kurtosis values were first examined to assess whether the data approximated a normal distribution. The skewness values ranged from −0.687 to −0.070, while the kurtosis values ranged from −1.09 to 0.651. These values indicate that the scale scores were within acceptable limits for normality (Hair et al., 2019), thereby supporting the use of subsequent parametric analyses. Internal consistency was then assessed using Cronbach's alpha coefficients. The coefficients were 0.94 for the Attitudes Toward Sport Scale, 0.93 for the Academic Motivation Scale, and 0.78 for the Academic Life Satisfaction Scale, indicating acceptable to high internal consistency across all three instruments (George and Mallery, 2003). The adequacy of the measurement instruments was further evaluated through confirmatory factor analysis. For the Attitudes Toward Sport Scale, the fit indices were χ^2^/df = 3.65, CFI = 0.89, RMSEA = 0.08, and SRMR = 0.06, indicating an acceptable level of fit. For the Academic Motivation Scale, the fit indices were χ^2^/df = 3.46, CFI = 0.85, RMSEA = 0.07, and SRMR = 0.08, which also suggest acceptable fit, although the CFI value was at the lower bound of acceptability. For the Academic Life Satisfaction Scale, the fit indices were χ^2^/df = 3.06, CFI = 0.97, RMSEA = 0.07, and SRMR = 0.04, indicating good model fit overall. Taken together, these results suggest that the instruments used in the study demonstrated acceptable psychometric properties for the subsequent analyses (Byrne, 2016; Gürbüz, 2021; Kline, 2023). Descriptive statistics and Pearson correlation coefficients for the study variables are presented in Table 4. As demonstrated in Table 5, the proposed multi-factor measurement model exhibited a significantly better fit to the observed data compared to the single-factor model. Specifically, the chi-square to degrees of freedom ratio (*x*^2^/*df* ), along with other fit indices (CFI, TLI, and RMSEA), indicated that the three-factor structure (ATS, AM, and ALS) provides a superior representation of the study variables. These results provide empirical evidence that the study constructs are conceptually distinct, thereby mitigating concerns regarding potential common method bias.

**Table 3:** Statistical model fit indices and psychometric properties.

Index	Good Fit	Acceptable	ATS	AM	ALS
*χ^2^/df*	≤ 3	3 < *χ^2^/df* ≤ 5	3.65	3.46	3.06
CFI	≥0.95	≥0.85	0.89	0.85	0.97
RMSEA	≤ 0.05	≤ 0.08	0.08	0.07	0.07
SRMR	≤ 0.05	≤ 0.08	0.06	0.08	0.04
Cronbach's alpha	≥0.70	≥0.60	0.94	0.93	0.78
Skewness	−1 to +1	−2 to +2	−0.070	−0.687	−0.222
Kurtosis	−1 to +1	−2 to +2	−1.09	0.651	0.137

ATS, Attitudes Toward Sport; AM, Academic Motivation; ALS, Academic Life Satisfaction.

**Table 4:** Means, standard deviations, and correlations among the study variables.

Variables	ATS	AM	ALS	*M*	SD
ATS	1.00			94.44	9.90
AM	0.42^**^	1.00		129.75	17.84
ALS	0.19^**^	0.43^**^	1.00	28.63	5.59

ATS, Attitudes Toward Sports; AM, Academic Motivation; ALS, Academic Life Satisfaction. ^**^p < 0.01

**Table 5:** CFA-based comparison of measurement models.

Model	*X* ^2^	*df*	*X*^2^/*df*
Proposed multi-factor model	*5,005*	*1,374*	*3.64*
Single-factor model	7,935	*1,377*	*5.76*

The proposed multi-factor model included three latent factors (ATS, AM, and ALS). The single-factor model was a constrained model where all items were forced to load onto a single latent factor.

As shown in Table 4, attitudes toward sports were positively associated with both academic motivation (*r* = 0.42, *p* < 0.01) and academic life satisfaction (*r* = 0.19, *p* < 0.01). Academic motivation was also positively associated with academic life satisfaction (*r* = 0.43, *p* < 0.01). These findings indicate that higher levels of attitudes toward sports are related to higher levels of academic motivation and academic life satisfaction, and that academic motivation is positively related to academic life satisfaction. The mean scores were 94.44 (SD = 9.90) for ATS, 129.75 (SD = 17.84) for AM, and 28.63 (SD = 5.59) for ALS. These correlation patterns provide initial support for the hypothesized relationships among the variables.

### 3.1 Indirect association analysis

**Figure 2:**
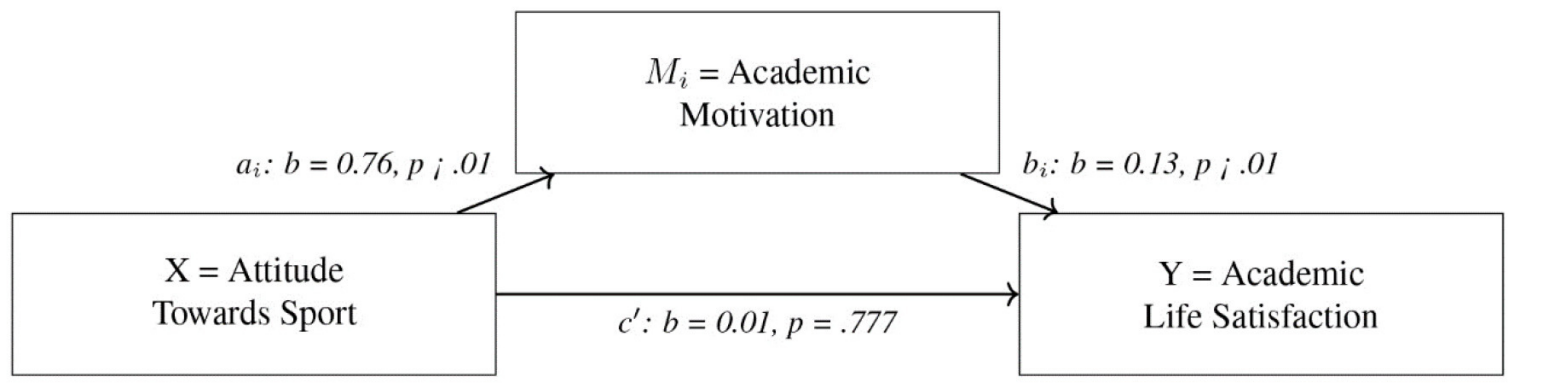
Indirect association model illustrating the direct and indirect statistical relationships among attitudes toward sports (X), academic motivation (M), and academic life satisfaction (Y).

The indirect association model was examined to evaluate whether academic motivation statistically accounted for the association between attitudes toward sports and academic life satisfaction. The results of the model are presented in Figure 2.

As illustrated in Figure 2, attitudes toward sports were positively associated with academic motivation, and academic motivation was positively associated with academic life satisfaction. This pattern indicated a statistically significant indirect association between attitudes toward sports and academic life satisfaction involving academic motivation. The direct association between attitudes toward sports and academic life satisfaction was not statistically significant when academic motivation was included in the model. The regression results corresponding to the model are presented in Tables 6–8.

**Table 6:** Regression results for attitudes toward sports predicting academic motivation (X → M) controlling for gender and age.

Variable	*B*	SE	*t*	*p*	95% CI Lower	95% CI Upper
Constant	49.01	9.58	5.12	< 0.001	30.19	67.84
ATS	0.80	0.08	10.10	< 0.001	0.64	0.95
Gender	5.45	1.56	3.49	0.001	2.38	8.52
Age	−0.22	0.29	−0.76	0.447	−0.78	0.34

ATS, Attitudes Toward Sports. *R* = 0.447, *R*^2^ = 0.200, *F*(3, 430) = 35.73, *p* < 0.001.

**Table 7:** Regression results for attitudes toward sports and academic motivation predicting academic life satisfaction (X, M → Y) controlling for gender and age.

Variable	*B*	SE	*t*	*p*	95% CI lower	95% CI upper
Constant	10.50	3.12	3.37	0.001	4.37	16.62
ATS	−0.00	0.03	−0.06	0.955	−0.06	0.05
AM	0.14	0.02	8.96	< 0.001	0.11	0.17
Gender	−0.66	0.50	−1.31	0.191	−1.64	0.33
Age	0.07	0.09	0.81	0.416	−0.10	0.25

ATS, Attitudes Toward Sports; AM, Academic Motivation. *R* = 0.435, *R*^2^ = 0.189, *F*(4, 429) = 25.02, *p* < 0.001.

**Table 8:** Regression results for the total association between attitudes toward sports and academic life satisfaction (X → Y) controlling for gender and age.

Variable	*B*	SE	*t*	*p*	95% CI lower	95% CI upper
Constant	17.19	3.29	5.22	< 0.001	10.71	23.66
ATS	0.11	0.03	3.95	< 0.001	0.05	0.16
Gender	0.09	0.54	0.16	0.870	−0.97	1.14
Age	0.04	0.10	0.45	0.655	−0.15	0.24

ATS, Attitudes Toward Sports. *R* = 0.193, *R*^2^ = 0.037, *F*(3, 430) = 5.56, *p* = 0.001.

The results presented in Table 6 indicate that attitudes toward sports were positively and significantly associated with academic motivation after controlling for gender and age (*B* = 0.80, *p* < 0.001). Gender was also a significant predictor of academic motivation (*B* = 5.45, *p* = 0.001), whereas age was not significantly associated with academic motivation (*B* = −0.22, *p* = 0.447). The model explained 20.0% of the variance in academic motivation (*R*^2^ = 0.200).

The results presented in Table 7 show that the overall model was statistically significant and explained 18.9% of the variance in academic life satisfaction (*R*^2^ = 0.189). Academic motivation was positively and significantly associated with academic life satisfaction (*B* = 0.14, *p* < 0.001). In contrast, the direct association between attitudes toward sports and academic life satisfaction was not statistically significant after controlling for academic motivation, gender, and age (*B* = −0.00, *p* = 0.955). Neither gender nor age was significantly associated with academic life satisfaction. These findings are consistent with the possibility that academic motivation statistically accounts for the association between attitudes toward sports and academic life satisfaction.

As shown in Table 8, attitudes toward sports were significantly associated with academic life satisfaction before academic motivation was included in the model, even after controlling for gender and age (*B* = 0.11, *p* < 0.001). The model explained 3.7% of the variance in academic life satisfaction (*R*^2^ = 0.037). Neither gender nor age was significantly associated with academic life satisfaction in the total effect model. To further evaluate whether this pattern was consistent with an indirect association involving academic motivation, the bootstrap estimate of the indirect association is presented in Table 9.

**Table 9:** Indirect association between attitudes toward sports and academic life satisfaction involving academic motivation (X → M → Y).

Path	Estimate	SE	95% CI Lower	95% CI Upper
ATS → AM → ALS	0.109	0.019	0.075	0.148

Bootstrap 95% confidence intervals are based on 5,000 resamples. Age and gender were included as covariates in the model. The indirect estimate is interpreted as a statistical indirect association due to the cross-sectional design of the study.

The results presented in Table 8 indicate a statistically significant indirect association between attitudes toward sports and academic life satisfaction involving academic motivation [effect = 0.109, BootSE = 0.019, 95% CI (0.075, 0.148)]. Because the confidence interval did not include zero, the indirect association was considered statistically significant. When considered alongside the non-significant direct association reported in Table 6, these findings suggest that academic motivation functions as an important statistical linking variable between attitudes toward sports and academic life satisfaction.

To control for potential demographic influences, age and gender were included as covariates in the mediation model. In the prediction of academic motivation, attitudes toward sports remained a significant positive predictor (*B* = 0.80, *p* < 0.001), while gender was also significant (*B* = 5.45, *p* = 0.001). Age was not significantly associated with academic motivation (*B* = −0.22, *p* = 0.447). In the prediction of academic life satisfaction, academic motivation was a significant positive predictor (*B* = 0.14, *p* < 0.001), whereas neither gender (*B* = −0.66, *p* = 0.191) nor age (*B* = 0.07, *p* = 0.416) showed significant effects. The direct association between attitudes toward sports and academic life satisfaction remained non-significant after controlling for academic motivation, age, and gender [*B* = −0.002, 95% CI (−0.056, 0.053), *p* = 0.955]. However, the indirect association through academic motivation remained statistically significant [effect = 0.109, BootSE = 0.019, 95% CI (0.075, 0.148)].

## 4 Discussion and conclusion

This study examined the associations among attitudes toward sports, academic motivation, and academic life satisfaction, with particular attention to whether academic motivation statistically accounted for the relationship between attitudes toward sports and academic life satisfaction. Overall, the findings supported the proposed associations and highlighted the importance of academic motivation as a statistical linking variable in understanding how attitudes toward sports are related to academic life satisfaction.

The findings supported this hypothesis, showing that students with more positive attitudes toward sports tended to report higher levels of academic motivation. One possible explanation is that positive attitudes toward sports may be associated with forms of engagement related to self-regulation, goal orientation, and a sense of competence, which are closely related to academic motivation. From the perspective of Self-determination Theory, sport-related experiences may be associated with autonomy and competence, which are closely related to students' motivation toward learning (Deci and Ryan, 2000). This interpretation is in line with previous research suggesting that engagement in sports is positively related to academic motivation and effort (Alahmed et al., 2016; Yukhymenko-Lescroart, 2021). Similarly, other studies have highlighted the role of self-discipline and goal orientation in linking sports-related experiences with academic functioning (Fortes et al., 2010; Hart et al., 2024). Overall, these findings suggest that students who develop more positive attitudes toward sports may also be more motivated in their academic lives.

The findings indicated that attitudes toward sports and academic motivation were positively associated with academic life satisfaction. Students with more positive attitudes toward sports reported greater academic life satisfaction at the bivariate level, while students with higher academic motivation consistently demonstrated more positive evaluations of their academic experiences. These findings are consistent with previous studies showing that sport participation and positive sport-related attitudes are associated with school satisfaction, life satisfaction, and academic adjustment (Belli et al., 2019; Caz et al., 2019; Slavinski et al., 2021; Kuśnierz et al., 2020; Yurdakul, 2020). Likewise, previous research has shown that academic motivation is positively related to academic satisfaction in higher education settings (Morelli et al., 2023; Wach et al., 2016; Puklek Levpušček and Podlesek, 2019; Abdi et al., 2025).

Academic motivation may contribute to academic life satisfaction because motivated students are generally more willing to invest effort, pursue meaningful goals, and remain engaged in the learning process (Pintrich, 2003). These characteristics are likely to facilitate more positive evaluations of academic experiences. Erdogan et al. (2021) similarly emphasized that university students' prospective wellbeing and active life perceptions contribute to adjustment and satisfaction within higher education settings.

The findings supported the hypothesis that academic motivation statistically accounted for the indirect association between attitudes toward sports and academic life satisfaction. Although the direct association between attitudes toward sports and academic life satisfaction was not statistically significant after academic motivation was included in the model (*B* = 0.01, *p* = 0.777), the indirect association involving academic motivation was positive and statistically significant [effect = 0.1007, 95% CI (0.0693, 0.1358)]. However, because the study used a cross-sectional design, these findings should not be interpreted as evidence of causal mediation or temporal ordering.

One of the most noteworthy findings was that the initially positive association between attitudes toward sports and academic life satisfaction disappeared after academic motivation was taken into account. This pattern suggests that positive attitudes toward sports may not directly enhance students' satisfaction with academic life. Rather, their influence appears to operate through motivational processes that support learning and engagement. In other words, holding favorable attitudes toward sports alone may not be sufficient to promote positive academic experiences unless these attitudes are accompanied by stronger academic motivation.

From a theoretical perspective, this finding is consistent with Self-determination Theory, which proposes that the benefits derived from experiences are not attributable merely to participation itself but to the extent that these experiences satisfy psychological needs and foster autonomous motivation (Deci and Ryan, 2000). Positive attitudes toward sports may be associated with feelings of competence, self-regulation, and goal orientation, which subsequently strengthen academic motivation. Therefore, academic motivation appears to represent a more proximal determinant of academic life satisfaction, whereas attitudes toward sports may function as a more distal factor. This distinction has important theoretical implications. It suggests that experiences in one life domain do not necessarily exert direct effects on outcomes in another domain. Instead, their effects may depend on shared psychological mechanisms. Thus, sport-related attitudes become academically meaningful not because they directly increase academic life satisfaction, but because they are associated with motivational resources that support engagement, persistence, and meaningful involvement in academic activities.

This interpretation is broadly consistent with previous research. Studies have shown that sport-related engagement is associated with stronger motivation and more positive academic functioning, while academic motivation itself has been linked to academic satisfaction through factors such as self-efficacy, engagement, and perceived competence (Jeno et al., 2023; Lepper et al., 2005; Alahmed et al., 2016; Pham et al., 2024; Vasconcellos et al., 2020). Related findings also suggest that autonomy-supportive and competence-enhancing environments may strengthen students' motivation and academic adjustment (Amorose and Anderson-Butcher, 2007; Jeno et al., 2018; Kusurkar et al., 2011). Similarly, Choi and Smith (2024) reported that students who viewed sport as an area of learning and development tended to demonstrate stronger motivation. Taken together, these findings support a more integrated understanding of student development. Rather than viewing attitudes toward sports, academic motivation, and academic life satisfaction as isolated constructs, the present study suggests that they are interconnected through motivational processes. More broadly, the findings imply that experiences originating outside the academic domain may contribute to students' academic wellbeing when they foster psychological resources that are relevant to learning.

### 4.1 Limitations

Several limitations should be considered when interpreting the findings of this study. First, the study relied on a cross-sectional design, which means that the observed relationships should be interpreted in associational rather than causal terms. Although the findings supported the proposed indirect association model, they do not provide evidence of temporal ordering, causal direction, or causal mediation. Therefore, the indirect association involving academic motivation should be understood as a statistical pattern rather than as evidence that attitudes toward sports influence academic life satisfaction through academic motivation over time. Second, the study was conducted with undergraduate students from a single public university using a non-probability sampling approach. In addition, the sample was limited to students from non-sport academic programs. These characteristics may limit the extent to which the findings can be generalized to students from other institutional contexts, age groups, educational levels, or sport-related academic fields. Third, all data were collected through self-report measures, which may have increased the risk of response bias, including social desirability and shared method-related bias. Finally, although the sample size was adequate for the analyses conducted, future research would benefit from longitudinal or experimental designs, more diverse and multi-institutional samples, and the inclusion of additional contextual or behavioral indicators to test whether the indirect associations observed in this study are replicated over time. In addition, one item in the Academic Motivation Scale demonstrated a relatively low standardized factor loading. Although the overall composite reliability of the scale remained satisfactory (CR = 0.91) and the original validated scale structure was retained, this issue should be considered when interpreting the measurement properties of the construct.

### 4.2 Implications

Future research may build on the present model by including variables such as self-regulation, psychological resilience, or academic commitment in order to develop a more detailed understanding of the statistical and theoretical links among attitudes toward sports, academic motivation, and academic life satisfaction. It would also be valuable to examine whether these relationships remain similar across different cultural or institutional contexts, as the role of sport and motivation may vary depending on broader social and educational conditions. From a practical perspective, the findings suggest that the academic relevance of positive attitudes toward sports may depend largely on the motivational resources associated with these attitudes. Accordingly, educational institutions may benefit from creating environments that support students' voluntary participation in sport and physical activity while simultaneously fostering motivational processes that encourage engagement with academic activities. Such environments may contribute to students' overall academic wellbeing by supporting both physical and psychological dimensions of student development.

## 5 Conclusion

This study examined the associations among attitudes toward sports, academic motivation, and academic life satisfaction and found that these variables were significantly related. In particular, the findings indicated that the association between attitudes toward sports and academic life satisfaction was better understood when academic motivation was included as a statistical linking variable, rather than as a direct association alone. One of the main contributions of the study is that it highlights the importance of motivational processes in explaining how experiences originating outside the academic domain may be related to students' academic wellbeing. The findings suggest that positive attitudes toward sports become academically meaningful primarily when they are accompanied by stronger motivation toward learning and engagement in academic activities. From the perspective of Self-determination Theory, these findings support the view that psychological and motivational resources may represent common mechanisms linking different domains of students' experiences. Thus, rather than viewing sport solely as a physical or extracurricular activity, the present study suggests that sport-related attitudes may have broader relevance within the academic context through their association with academic motivation. Overall, the findings provide a more integrated understanding of the relationships among attitudes toward sports, academic motivation, and academic life satisfaction in higher education settings. Given the cross-sectional design of the study, these conclusions should be interpreted in terms of statistical associations rather than causal mediation.

### 5.1 Recommendations

- Higher education institutions may consider developing initiatives that foster positive attitudes toward sports while simultaneously supporting students' academic engagement. Such initiatives may help students connect positive sport-related experiences with broader educational goals.
- Student support services may emphasize the potential motivational value of positive sport-related attitudes. Helping students reflect on experiences associated with discipline, goal orientation, and personal development may contribute to greater academic engagement.
- Universities may create opportunities that encourage positive perceptions of sport and physical activity as part of a balanced student experience. Such initiatives may contribute to students' overall academic wellbeing through motivational processes.
- Academic advisors and student development professionals may encourage students to recognize how personal interests, including sport-related interests, are associated with motivation, persistence, and engagement in academic activities. Future interventions should focus on strengthening these motivational resources rather than assuming direct effects on academic outcomes.

## Data Availability

The original contributions presented in the study are included in the article/supplementary material, further inquiries can be directed to the corresponding author.
